# Free-breathing respiratory self-gated Golden angle RAdial Three-dimensional whole-heart isotropic cine imaging for left ventricular volumetric Evaluation (GRATE) - comparison with conventional 2D breath-hold cine imaging

**DOI:** 10.1186/1532-429X-18-S1-P242

**Published:** 2016-01-27

**Authors:** Karen Holst, Martin Ugander, Andreas Sigfridsson

**Affiliations:** grid.24381.3c0000000092415705Department of Clinical Physiology, Karolinska Institutet and Karolinska University Hospital, Stockholm, Sweden

## Background

Breath holding during imaging can be challenging for patients with heart disease and can affect image quality as well as alignment of images acquired during different breath holds. The purpose of this study was to develop a free breathing respiratory self-gated cardiac magnetic resonance (CMR) technique for left ventricular (LV) volume measurements.

## Methods

***Acquisition.*** A balanced steady-state free precession (bSSFP) sequence using a 3-dimensional (3D) golden angle radial acquisition order was implemented for acquisition of an image volume covering the whole heart (GRATE). The sequence was used to image 8 healthy volunteers during free breathing and over several cardiac and respiratory cycles. A stack of short-axis (SA) slices, as well as 4-, 3- and 2-chamber views, were acquired with a conventional Cartesian 2-dimensional (2D) breath held cine bSSFP for comparison. Both methods were applied twice on each subject with a break outside the scanner in between. GRATE parameters were: FOV 352 mm, isotropic voxels 2 × 2 × 2 mm, TR/TE 2.7/1.3 ms, flip angle 65°, 900,000 radial spokes.

***Respiratory Self-gating.*** Respiratory self-gating (SG) signals from the data were extracted retrospectively from the *k*-space center point in each radial spoke using a two-sided low-pass filter adapted to each dataset. Trigger points for each respiratory cycle were derived from the angle of the analytic signal, i.e. filtered using a quadrature low-pass filter that only kept positive frequencies.

***Image reconstruction.*** The respiratory SG signal and recorded ECG were used to bin each radial spoke into 3 respiratory phases and 25 cardiac phases. For whole heart image reconstruction of each bin, selected spokes from neighboring bins were also used, chosen to give a more evenly distributed *k-*space sampling. From each image volume multi-planar reformatting was used to extract a SA stack with the same resolution as the breath held SA stack (2 × 2 × 8 mm). LV volumes were obtained from manual segmentation of each SA stack. Data are given as mean ± SD.

## Results

The number of radial spokes in each unique cardiac and respiratory phase combination over all subjects was 25,165 ± 137. There was no difference between GRATE and 2D with regards to LV ejection fraction (EF) (60 ± 4 % vs. 61 ± 4 %, p = 0.38) or stroke volume (SV) (109 ± 21 ml vs. 116 ± 24 ml, p = 0.08). The test re-test difference did not differ between GRATE and 2D for either EF (2 ± 2 % vs. 1 ± 4 %, p = 0.55) or SV (1 ± 10 ml vs. 1 ± 9 ml, p = 0.64). The difference between GRATE and 2D measurements was 1 ± 4 % for EF, and 7 ± 10 ml for SV.

## Conclusions

GRATE and conventional 2D cine showed similar absolute values and test re-test repeatability for LVEF and SV in healthy volunteers. 3D golden angle radial acquisition during free breathing enabled retrospective sorting of the data from respiratory self-gated signals and ECG, and could correctly measure LV volumes. Furthermore, GRATE imaging allows for unrestricted multiplanar reformatting after acquisition.

**Figure 1:**
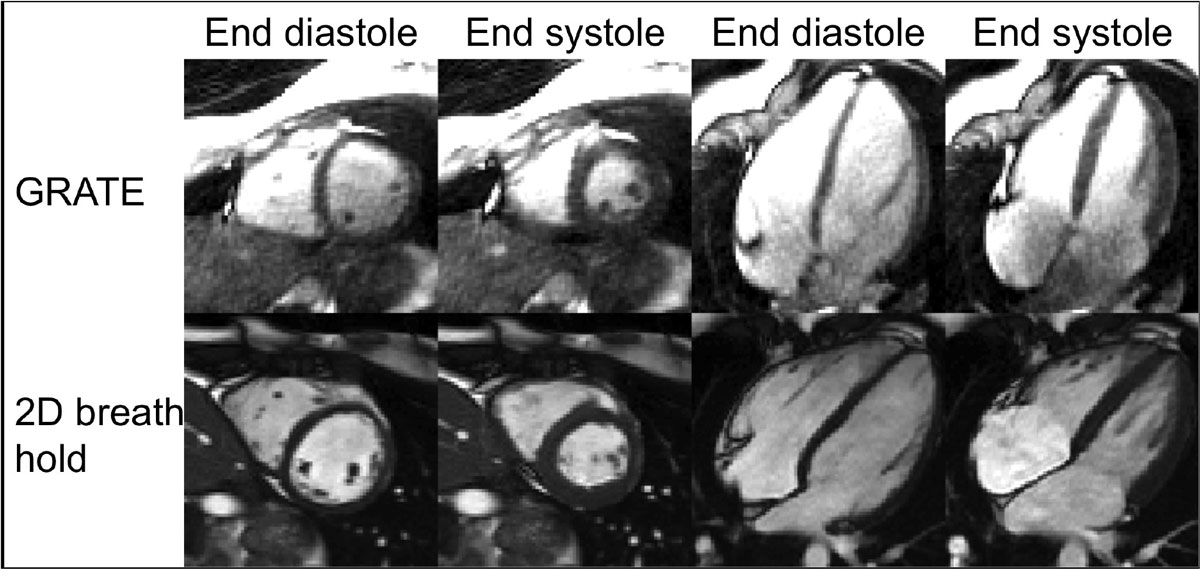
Figure 1

